# Single-molecule visualization of mRNA circularization during translation

**DOI:** 10.1038/s12276-023-00933-1

**Published:** 2023-01-31

**Authors:** Byungju Kim, Jincheol Seol, Yoon Ki Kim, Jong-Bong Lee

**Affiliations:** 1 Department of Physics, Pohang University of Science & Technology (POSTECH), Pohang, 37673 Republic of Korea; 2 School of Interdisciplinary Bioscience and Bioengineering, POSTECH, Pohang, 37673 Republic of Korea; 3 Department of Biological Sciences, Korea Advanced Institute of Science and Technology, Daejeon, 34141 Republic of Korea

**Keywords:** Single-molecule biophysics, Ribosome

## Abstract

Translation is mediated by precisely orchestrated sequential interactions among translation initiation components, mRNA, and ribosomes. Biochemical, structural, and genetic techniques have revealed the fundamental mechanism that determines what occurs and when, where and in what order. Most mRNAs are circularized via the eIF4E–eIF4G–PABP interaction, which stabilizes mRNAs and enhances translation by recycling ribosomes. However, studies using single-molecule fluorescence imaging have allowed for the visualization of complex data that opposes the traditional “functional circularization” theory. Here, we briefly introduce single-molecule techniques applied to studies on mRNA circularization and describe the results of in vitro and live-cell imaging. Finally, we discuss relevant insights and questions gained from single-molecule research related to translation.

## Protein synthesis: Seeing single mRNA molecules

Single-molecule fluorescence imaging is revealing the formation of circular mRNA and the complex interplay of messenger RNA (mRNA) and ribosomal proteins during translation, the decoding of mRNA to form proteins. Various translation factors encourage the circularization of mRNA, which might help control mRNA expression, but detailed insights have been limited. Jong-Bong Lee at Pohang University of Science & Technology, South Korea, and co-workers reviewed recent progress in single-molecule imaging of mRNA circularization. Various fluorescence imaging methods have revealed the changing shapes, structures, locations and binding kinetics of mRNA molecules, as well as the quantities of important target proteins. The researchers highlight future challenges, such as simultaneously tracking multiple translation regulators, and improving protein purification and labeling. Such detailed single-molecule visualization could eventually enable scientists to directly manipulate interactions within mRNA-protein complexes.

## Introduction

In protein synthesis, translation initiation is a rate-limiting step because of cascading mRNA and protein (mRNP) formation. Extensive research has revealed that this process is regulated by more than 12 translation initiation factors^1,2^. During the process, mRNA undergoes functional circularization (or becomes a “closed loop”) through protein–mRNA and protein–protein interactions to positively regulate protein synthesis^3,4^; that is, functional circularization^5^ facilitates the recycling of posttermination ribosomes for reinitiation^6,7^ and shields the mRNA from decay factors^8,9^. Since all eukaryotic mRNAs, except histone mRNA, have a unique m7G cap structure at the 5ʹ-end and a poly(A) tail at the 3ʹ-end, circularization mostly begins with the recognition of binding proteins to both ends, namely, 5ʹ-cap-eIF4E^2,10^ and 3ʹ-poly(A) tail-PABP^11^, respectively. Some mRNAs utilize an internal ribosome entry site (IRES)^12,13^, N^6^-methyladenosine (m^6^A)^14,15^, or elements related to viruses^16–18^ instead of a cap and/or poly(A) tail to interact with their binding partners. Each binding protein can communicate with other binding proteins at the other end with the help of mediators such as eIF4G^19,20^ and/or eIF3^15^. Moreover, since circularization is based on stochastic interactions with biomolecules, it may not result in a fixed loop; instead, the loop can change dynamically, suggesting that kinetic/dynamic studies on mRNP components during translation contribute to an integrated understanding of translation^21^. However, because of technical difficulties, which involve the fluorescent labeling of translation factors, single-particle tracking (SPT) in high-background signals, and spatiotemporal resolution limits, only direct structural evidence has been obtained through atomic force microscopy^22^. Thus, mRNA circularization remains poorly understood.

Excitingly, single-molecule fluorescence imaging (smFI) has been employed to examine mRNA circularization in live cells, which are composed of numerous transitory interactions^23,24^. Conformational changes in mRNA are visualized by SPT with high spatiotemporal resolution. It has enabled us to determine the relationship between translation and circularization in cells. Meanwhile, in vitro sm studies on initiation factors have revealed their binding kinetics or conformational change in protein synthesis, thus contributing significantly to our understanding of the molecular processes involved in circularization^25–29^. In this review, we briefly introduce how smFI techniques have been applied to investigate mRNA circularization and discuss the current knowledge of the relationship between translation and mRNA circularization. Finally, to provide perspective, we address some questions arising from these sm studies.

### smFI techniques applied to mRNA circularization studies

The movement of a regulator or mRNA should be explored with high spatiotemporal resolution, or the end-to-end distance should be measured directly to examine functional circularization from interactions between translation factors and/or mRNA. The following smFI techniques are frequently used to obtain these parameters.

#### smFRET

**Fig. 1:**
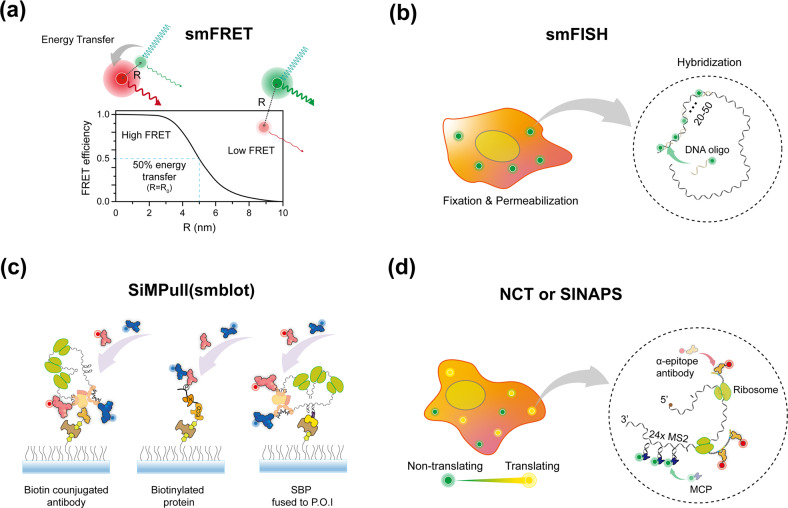
SmFI techniques applied to an mRNA circularization study. **a** The relationship between FRET efficiency and interdye distance. When the interdistance R between the donor (green) and acceptor (red) is within 10 nm, the excited energy of the donor is transferred to the acceptor, which subsequently emits fluorescence. The FRET efficiency is inversely proportional to the interdye distance, and 50% of the energy is transferred when R is equal to R_0_. **b** Principle of smFISH. Cells are fixed and permeabilized for introducing the probes (fluorescently labeled DNA oligonucleotides with ~20 nt, which is a sufficient length for stable hybridization with RNA at room temperature). Typically, 20–50 probes are used for signal amplification. **c** Schematic showing the mRNP complex or EGFR immobilized on a PEG-biotin surface via either a biotin-conjugated antibody, biotinylated protein, or SBP fused to the protein of interest. A fluorescently labeled antibody is introduced that binds to the target. The heterogeneous state of a single mRNP or EGFR is investigated quantitatively, including the number or combination of constituents. **d** Nontranslating (green) or translating (yellow) mRNAs in a cell. As the ribosome synthesizes the epitopes in a coding sequence, the fluorescently labeled antibody (red) binds to the nascent epitope, and the fluorescence intensity depends on the number of nascent epitopes. Fluorescently labeled MCP binds to the MS2 stem‒loop in the 3ʹ-UTR, irrespective of the translational state, and indicates the location of the mRNA.

Single-molecule Förster resonance energy transfer (smFRET) is one of the most widely used imaging techniques in the field. It examines the interaction between molecules or the interdistance of residues in a molecule by measuring the efficiency of transferred nonradiative energy from one fluorophore (donor) to another^30,31^ (acceptor; Fig. 1a). Because energy transfer occurs when two fluorophores are within the range of several nanometers (1–10 nm), i.e., it rapidly changes within the Förster distance (R_0_), the strength and length of interactions can be determined in real time by taking advantage of the distance-sensitive feature^32,33^. This technique has been applied to investigate the binding kinetics or dynamic conformation changes in solutions.

#### smFISH

Single-molecule fluorescence in situ hybridization (smFISH) is a technique used to detect and localize single mRNAs in a cell by using dye-labeled DNA probes^34,35^ (Fig. 1b). DNA probes are designed to have a sequence complementary to their mRNA partner, with a 17–22-mer probe used for hybridization. Since smFISH relies on hydrogen bonding between DNA and mRNA, 20–50 probes per transcript are typically used to achieve a sufficient number of base pairs and obtain bright fluorescence signals for high-precision localization. One advantage of smFISH is that even without modifying the target mRNA, the native status of mRNAs in a cell can be determined during translation. Thus, smFISH has been applied to measure the end-to-end distance of mRNAs due to the free labeling positions.

#### SiMPull

In contrast to conventional blotting assays that show an ensemble-averaged state, the single-molecule pull-down (SiMPull) assay is useful for investigating the heterogeneous state of a target protein complex^36,37^ (Fig. 1c). Unlike conventional blotting, the target is immobilized on the surface via a specific molecule (antibody^36,37^, biotin^38^, or streptavidin-binding peptide^39^ [SBP]) and detected using fluorescently labeled antibodies. A fluorescence signal indicates whether a specific component is contained in a target. When SiMPull is conducted with multicolor imaging, the combination of fluorescence signals provides the quantitative ratio of components.

#### NCT or SINAPS

Nascent chain tracking (NCT) and single-molecule imaging of nascent peptides (SINAPS) are novel techniques that visualize nascent proteins synthesized from mRNA in cells via a multimeric array system^24,40–43^ (Fig. 1d). A reporter mRNA contains two multimeric arrays in different regions: a repetitive MS2 or PP7 stem‒loop array on the mRNA 3ʹ-UTR and repetitive epitope-coding sequences in an open reading frame. A single mRNA can be visualized by fluorescently labeling the MS2 coat protein (MCP) or PP7 coat protein (PCP), which bind to the MS2 and PP7, respectively. A fluorescence signal is amplified through a repetitive array, which enables the mRNA to be separated from a background signal. Similar to the MS2 or PP7 system, translation with a single mRNA is visualized by using a fluorescently labeled antibody (Fab or scFv) that binds to its epitope. As a ribosome translates the epitope region, the fluorescence signal is increased because more nascent epitopes can be bonded to the antibody. Thus, the location of mRNA and the status of translation in living cells can be visualized using multimeric array systems.

### smFI studies in mRNA circularization

Studies have examined mRNA circularization from purified systems to living cells. However, direct observations (end-to-end distance of mRNP) in cells have only been made recently, with several sm studies instead focusing on the association/dissociation or conformational change in these factors and/or mRNA. Here, we describe some aspects related to circularization.

#### mRNA circularization studies involving purified systems

**Fig. 2:**
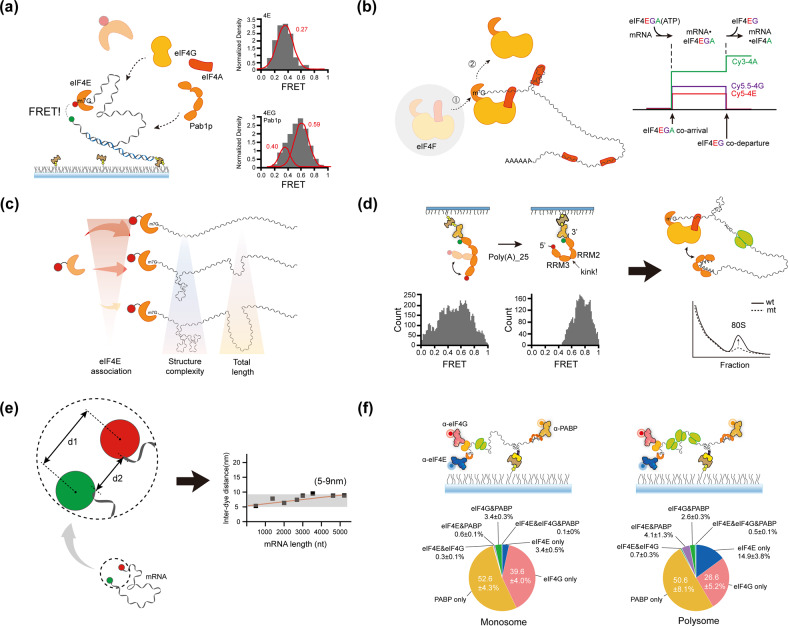
Sm mRNA circularization studies in purified systems. **a** Schematic of the eIF4E-cap (RNA) binding kinetics in the absence or presence of other factors (eIF4G, eIF4A, and Pab1p) alongside the FRET value. Capped RNA is immobilized on the PEG-biotin surface via streptavidin-biotin interactions. FRET indicates that Cy5-eIF4E binds to the cap of Cy3-RNA. The FRET value increases in the presence of other factors. **b** Schematic and intensity-time trace representing the comprehensive eIF4F-cap (mRNA) binding kinetics during translation initiation. eIF4F (eIF4E, eIF4A and eIF4G) binds to the mRNA cap (coarrival). Shortly after (~0.08 s^-1^), eIF4E dissociates from mRNA with eIF4G (codeparture). Meanwhile, eIF4E-cap binding is enhanced by the interaction between “free” eIF4A and mRNA, independent of ATP hydrolysis. **c** The effect of structure and length of mRNA on eIF4E association, which depends on the mRNA length and complexity but not on the free energy. **d** Left: Schematic representation of an smFRET system that directly monitors the bent conformation of fluorescently labeled RRM1-4 in the absence (left) or presence (right) of poly(A)_25 RNA alongside a histogram of the FRET efficiency in the absence or presence of poly(A)_25 RNA. Right: Schematic figure representing the role of bent conformation in mRNA circularization for translation. It enhances the eIF4G–PABP interaction, enhancing the formation of 80S in an in vitro translation system. **e** Direct measurement of the end-to-end distance of mRNA. The interdistance of two dyes (d1) does not reflect the actual end-to-end distance (d2). The fluorophore linker length is considered to obtain an exact value, producing 5–9 nm among the investigated mRNAs. **f** Schematic of a single polysome tethering assay that visualizes the heterogeneous state of a single mRNP complex for a monosome (left) and a polysome (right). The components of the mRNP complex were captured by fluorescently labeled antibodies. A pie chart indicating that most mRNAs contain only one factor.

It is believed that circularization begins with the recognition of the 5ʹ-cap of mRNA by eIF4F (eIF4E, eIF4A, and eIF4G), in conjunction with other initiation factors, as the main part of initiation control^2^. Recognition of eIF4G increases eIF4E’s affinity to a cap or cap analog^44,45^. To study the 5’ cap-binding kinetics of eIF4E, smFRET was monitored with fluorescently labeled eIF4E in the absence and presence of eIF4G^25,46,47^ (Fig. 2a). smFRET experiments have demonstrated that full-length yeast eIF4G promotes an alternate conformational state of the RNP complex, causing it to reach the ends of an mRNA. Interestingly, Pab1p, a yeast PABP, has a more significant effect in causing both RNA ends to close even in the absence of poly(A) and/or eIF4G. This observation suggests that PABP may participate in the conformational changes of RNA by directly interacting with mRNA, although its interaction with mRNA itself is relatively weaker than its interaction with the poly(A) tail^48,49^. A follow-up study on the kinetics of the eIF4E–mRNA interaction has been performed more comprehensively using fluorescently labeled eIF4E, eIF4A, eIF4G, and mRNA immobilized on a surface^25^. Direct eIF4G–mRNA interactions rather than eIF4E–eIF4G interactions are the main cause for the acceleration of eIF4E-cap binding and the increased association time of eIF4E during translation initiation, which is consistent with a recent in vivo report suggesting that eIF4G may persist in mRNA and facilitate cap-binding activity^50^. Conversely, another smFRET study found that partial human eIF4G (557-1137) alone slightly affects eIF4E binding kinetics, but the human eIF4F complex substantially changes the eIF4E-cap association^46^. Interestingly, free eIF4A enhances eIF4E cap accessibility via direct eIF4A-RNA contact, which is similar to the role of eIF4G in eIF4E^25^. Meanwhile, the binding of eIF4F to RNA results in the dissociation of eIF4E from eIF4F shortly after the formation of the eIF4F-RNA complex, but this process is dependent on ATP hydrolysis (0.07–0.08 s^-1^; Fig. 2b). Notably, eIF4E and eIF4G are separated simultaneously rather than sequentially, which is consistent with an ensemble study on a yeast system; specifically, eIF4E and 4G preferentially leave mRNA during translation initiation–elongation transition^51^.

The effect of the structural diversity of mRNA^52^ or regulators^28,29^ on translation heterogeneities has recently been demonstrated. Indeed, translation is regulated by the secondary structure^53^, length^54,55^, mRNA sequence^56^, posttranscriptional modifications (e.g., m^6^A modification^57^), and poly(A) length^29^. Wang et al. demonstrated that the addition of a small stem loop (Δ*G* = − 4.8 kcal/mol) in the 5ʹ-UTR is sufficient to perturb the state in translation initiation and delay translation^58^, which is not detectable in an ensemble assay. The innate 5ʹ-UTR structure affects protein–mRNA dynamics^59^. The eIF4E–mRNA association is inversely proportional to the degree of secondary structure formation on the 5ʹ-UTR of the mRNA (Fig. 2c). Interestingly, the rate of eIF4E–mRNA association depends not on the free energy of the secondary structure but on the degree of complexity of the secondary structure. The rate of association between eIF4E and mRNA tends to increase as the total length of mRNA shortens, and the eIF4G–mRNA interaction accelerates the association rate of eIF4E in proportion to the length of mRNA; consequently, less deviation occurs among mRNAs^25^. Conformational changes in a regulator can play a crucial role in circularization. According to our smFRET study, conformational changes in PABP, specifically in the RRM2–RRM3 region, are induced when PABP binds to a poly(A) tail of RNA, and this conformational change affects PABP–poly(A)^29^ and PABP–eIF4G^28^ interactions (Fig. 2d). Mutation of the region inhibits the PABP–eIF4G interaction and inhibits the efficient formation of 80S ribosomes. Consistent with the canonical view, this result suggests the importance of PABP binding to the poly(A) tail in an end-proximity form of mRNA. Recently, this bent conformation of PABP was re-examined via cryo-EM^60^.

Despite structure-driven circularization, the direct measurement of the RNA end-to-end distance shows the inherent proximity of the mRNA ends. An smFRET study was conducted to experimentally determine the end-to-end distances of mRNAs of various lengths (500–5,500 nt) from viruses and fungi^61^ (Fig. 2e). mRNAs were labeled with a dye at each end, and the interdye distance was measured from the FRET efficiency. Interestingly, all mRNAs used in this study have an end-to-end distance within 5–9 nm without any protein, irrespective of length, origin, and secondary structure. Before this initial experimental demonstration, several theoretical calculations had estimated that the end-to-end distance of mRNA should be small and not dependent on mRNA length^62–64^. More recently, the inherent end-to-end distance was more systematically investigated with human mRNA and lncRNA^65^. Computational analysis and smFRET have shown that proximity is attributed not only to base pairing in the 5ʹ- or 3ʹ-UTR but also to stem loops formed in the whole sequence. Furthermore, even in the firefly luciferase ORF without a 5ʹ- and 3ʹ-UTR, the end-to-end distance was 4.9 nm. This inherent closeness of both ends of RNA in the absence of proteins is consistent with reports on cells described in the next section.

#### mRNA circularization in single polysomes

Many in vitro studies demonstrate functional circularization via translation factors. However, no direct investigation has focused on whether the circularized mRNP is formed via a 5ʹ-cap-eIF4E–eIF4G–PABP–poly(A) interaction in cells. Therefore, an sm blotting experiment was conducted using polysomal or subpolysomal fractions to find direct evidence of functional circularization^39^ (Fig. 2f). Immobilization of the cross-linked polysomal fraction to a PEG-biotin surface was performed using biotin-conjugated or fluorescently labeled antibodies against eIF4E, eIF4G or PABP. Strikingly, the results exhibited rare colocalization between factors in all fractions. Specifically, most mRNAs have only one member. Because endogenous mRNPs were cross-linked under normal conditions, this result suggested that mRNA circularization cannot occur in an active translation state. Furthermore, even in the case of an m^6^A-containing reporter mRNA, which was reported previously, the mRNA is more likely circularized through the interaction of the m^6^A reader protein YTHDF family^14^ or METTL3^15^ with eIFs; we could not find an increase in colocalization between factors^39^. Although a different m^6^A reporter was used, m^6^A-containing mRNA likely increases the translation efficiency, but it does not increase the level of mRNA circularization during translation.

#### mRNA circularization in cells

**Fig. 3:**
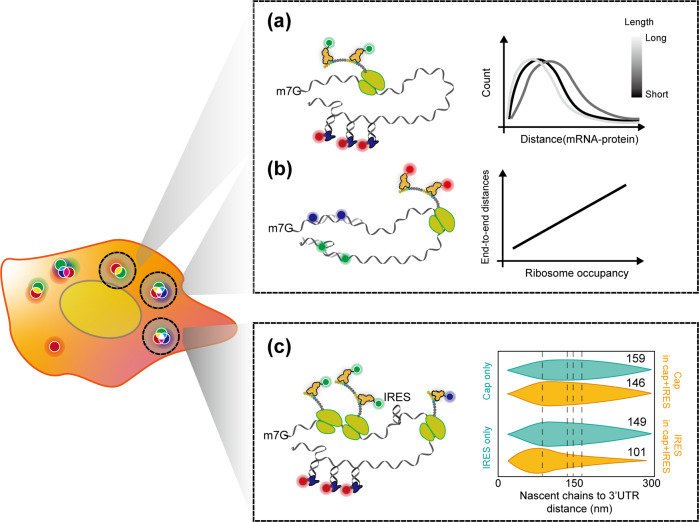
mRNA circularization in cells. Schematic representing nontranslating or translating mRNA and the measurement of its end-to-end distance in a cell (fixed or live). **a** The distance between the nascent epitope and the PP7 stem‒loop indicates the compactness of the translating mRNP. **b** Direct end-to-end distance measurement of mRNA during translation depending on the translation state through the utilization of a smFISH probe in a fixed cell. As ribosome occupancy increases, the distance between the two ends also increases. **c** End-to-end distance measurement of mRNA during cap or IRES translation (emerald) or both (orange). Cap translation leads to a longer distance than IRES translation resulting from ribosome occupancy.

Recent advances in single-RNA cellular imaging techniques have enabled the visualization of mRNA circularization during translation. Although it is primitive, the most direct method to investigate circularization is by measuring the distance of the mRNA compartment. Morisaki et al. quantified the size of polysomes with three different lengths (125, 374, and 1544 a.a.) by measuring the distance between nascent peptide chains and the 3ʹ-UTR of mRNA^24^ (Fig. 3a). Interestingly, NCT experiments have shown that the distance is shorter (65–105 nm) than expected, suggesting that polysomes are compact rather than extended. This is consistent with previous reports involving ET^66^ or EM^67^ (or cryo-EM) describing that polysomes have either a rosette^68,69^, helical^70^ or spiral shape^71^. However, the size was not correlated with the length of mRNA in this experiment. More recently, two groups reported a striking result: a closed loop is not a stable state of translating mRNA^72,73^. smFISH and SunTag signals were monitored under normal or stress (nontranslating) conditions to determine the architecture of mRNP (Fig. 3b). An smFISH experiment with a hybridizing probe at both ends of mRNA demonstrated that mRNP compaction depends on the translation state. More specifically, ribosome release is the main cause of compaction because compaction occurs in the 5ʹ- to 3ʹ-direction when translation initiation is inhibited^72^. Only under stress conditions, such as treatment with arsenite, heat, or translation inhibitors, does mRNP take a globular form, which is likely to cause functional circularization. The translating mRNP also possesses a compact (or globular) conformation, although to a lesser extent than under stress conditions. These findings are consistent with the results of the above NCT experiment; however, it is insufficient to cause both ends of the mRNA to connect. Interestingly, both studies have demonstrated the dependency of end-to-end distance on the ribosome occupancy of mRNA. Indeed, the longer the ORF, the greater the degree of ribosome elongation because the end-to-end distance is correlated with the length of mRNA. However, this result is different from that of the NCT experiment. Furthermore, the breakage of the eIF4G–PABP interaction does not lead to changes in compaction^73^, suggesting that the interaction between translation factors may not be a critical factor for the circularization of mRNA and that the proximity of the ends is an intrinsic feature of mRNA. This idea is also supported by the fact that long noncoding (lnc) RNA has compactness similar to that of nontranslating mRNA^65^. In addition, nuclear mRNA is slightly less compact than translationally inhibited mRNA because of the binding of RBPs^73^.

mRNA does not only undergo circularization when in the presence of eIF4E–eIF4G–PABP. Recently, Koch et al. demonstrated IRES-mediated mRNA compaction^23^. They constructed a reporter mRNA molecule that contains a cap and IRES downstream of the cap-ORF, which produced a 10x FLAG epitope and a 24x SunTag by the cap and IRES, respectively (Fig. 3c). In this study, the distances of the cap-ORF to 3ʹ-UTR and IRES-ORF to 3ʹ-UTR were measured in cap- or IRES-only translation or cap+IRES translation. Interestingly, the distance of the IRES-ORF to the 3ʹ-UTR was similar to that of the cap to the 3ʹ-UTR, suggesting that the IRES-ORF is compact when it is idle, possibly because of the lack of ribosomes on the IRES-ORF. The distance of ORF to 3ʹ-UTR in both cases is proportional to intensity, that is, the number of ribosomes, which is consistent with previous studies on fixed cells^72,73^. Furthermore, in the case of cap+IRES translation, the distance from the cap-ORF to the 3ʹ-UTR is slightly decreased, but the distance from the IRES-ORF to the 3ʹ-UTR is significantly decreased compared to cap- or IRES-only translation. This decrease occurs because there are fewer available ribosomes in cap+IRES translation than in cap- or IRES-only translation; the decreased number of ribosomes loaded on mRNA is represented by the distance decrease, which also supports the effect of ribosomes on mRNA compaction.

### Future perspectives

SmFI has provided an opportunity to examine mRNA circularization in view of the interaction between proteins and mRNAs, especially in relation to time. If translation or ribosome occupancy is the criterion for mRNA circularization, then how can recent sm data be reconciled with a considerable amount of ensemble data? Biochemically, circularization should occur on the basis of the binding affinities of protein–protein or protein–mRNA interactions and the concentration of proteins in cells^74,75^. During translation elongation, initiation also occurs. Then, how can ribosomes be reinitiated after termination? Does the communication of both ends of mRNA occur only in a nontranslating state? Is the extension of mRNA by loaded ribosomes sufficient to break the eIF4G–PABP or the eIF4E–eIF4G interaction? Or is there an unknown factor? Another interesting question is when functional circularization occurs. Indeed, a recent study has shown that the initiation rate increases only after the first ribosome has completed translation to capped and polyadenylated mRNA^3^. Here, it is possible that the first ribosome drags eIF4F and meets PABP at the 3ʹ-end, thus making the physical link. In addition to classical circularization via 5ʹ-cap-eIF4E–eIF4G–PABP-3ʹ-poly(A), mRNA circularization mediated by other structural IRESs^12,13^, m^6^A^14,15^, or 3’ cap-independent translational enhancers^16,76^ should be addressed for a comprehensive understanding. The smFI methods will help answer these questions. Since the development of NCT or SINAPS, the sm study of translational kinetics/dynamics in living cells has focused on various subjects^77–81^. Nevertheless, it is still difficult to track single or multiple translation regulators at the same time owing to the high intracellular concentration of the targets^74,75^, although one example has been reported^50^. Further advances in protein labeling strategies are also required beyond the several methods that have been developed, including small peptide tags and their cognate antibodies^82–84^, the incorporation of unnatural amino acids^85^ or chemical labeling without genetic manipulation^86^. Because of its high specificity and efficiency, the Halo-^87^ or SNAP-tag^88^ is still the preferred strategy despite the potential problems caused by the large tag size. Additionally, technical advances in protein purification are required for experiments in an in vitro system. Currently, to our knowledge, no purification of full-length human eIF4G has been reported^46^. In addition to the advancement of techniques, multiple approaches can be integrated. Recently, a study combined two methods, namely, fluorescence correlation spectroscopy (FCS) and SPT, to investigate cap-dependent translation initiation in living cells^50^. Each method complements the other’s shortcomings and reveals the temporal regulation of cap-binding activity at extraordinary resolutions.

## Concluding remarks

Functional mRNA circularization based on protein–protein or RNA–protein interactions has been considered a canonical dogma in translational studies. However, recent sm studies have shown that mRNA appears circularized, yet it cannot be physically connected by proteins in active translation. Although the smFI technique does not provide a complete solution, it is currently the most optimal method used for visualizing the dynamic behavior of single mRNP in intact cells. In the future, smFI may contribute to a comprehensive understanding of how we can change interactions in an mRNP complex and the resulting conformation of mRNA throughout translation.
